# Datasets of the phosphorus content in laundry and dishwasher detergents

**DOI:** 10.1016/j.dib.2018.11.081

**Published:** 2018-11-19

**Authors:** P.J.T.M. van Puijenbroek, A.H.W. Beusen, A.F. Bouwman

**Affiliations:** a PBL Netherlands Environmental Assessment Agency, PO Box 30314, The Hague, GH 2500, the Netherlands; b Department of Earth Sciences, Geochemistry, Faculty of Geosciences, Utrecht University, PO Box 80021, Utrecht, TA 3508, the Netherlands

## Abstract

This data article provides the data of Phosphorus emissions from laundry and dishwasher detergents as part of the Phosphorus emissions from households. The household emissions are presented in the research article “Global nitrogen and phosphorus in urban waste water based on the Shared Socio-economic pathway” (van Puijenbroek et al., 2019) [1]. Laundry and dishwasher detergents are a major source of phosphorus loading of aquatic ecosystems in countries with a substantial use of laundry and dishwasher machines.

In this article, datasets are presented with the global use of laundry and dishwasher detergents and the Phosphorus emissions due to laundry and dishwasher detergents. These results are presented for 10 world regions for 1970 and 2010, and for 2050 with 5 Shared Socio-economic Pathways. The outlook results for 2050 were based on the growth in income and population and on environmental policy for the introduction of Phosphorus free detergents.

**Specifications table**

**Table:** 

Subject area	*Chemistry and environmental policy*
More specific subject area	*Laundry detergents; Dishwasher detergents; phosphorus*
Type of data	*Figures, table*
How data was acquired	*Literature and policy targets*
Data format	*Analyzed and extrapolated*
Experimental factors	*The relationship between the use of laundry and dishwasher detergents and the national income per capita were determined*
Experimental features	*Assumptions of environmental policy to reduce the Phosphorus content in detergents were based on the storylines of the Socio-economic Pathways.*
Data source location	*Global dataset*
Data accessibility	*Data is with this article*
Related research article	*[1]**van Puijenbroek, P.J.T.M., A.H.W. Beusen, A.F. Bouwman, Global nitrogen and phosphorus in urban waste water based on the Shared Socio-economic pathways. J. Environ. Manag., 2019. 231: p. 446–456.*

**Value of the data**

• This dataset can be used for global estimates of phosphorus emissions due to laundry and dishwasher detergents.
• This dataset can be useful to show the effect of environmental policy on the discharges of phosphorus due to the use of detergents.

## 1 Data

The datasets of this article provides information on the use of Phosphorus in detergents ( Tables 1 and 2) and the emission of phosphorus due to the use of detergents ( Tables 3 and 4). Tables 1 and 2 show the Phosphorus use by laundry and dishwasher detergents, respectively, on a regional and a global basis per capita. Tables 3 and 4 showed the volume of the emissions of Phosphorus due to the use of laundry and dishwasher detergents.

**Table 1:** Use of Phosphorus in laundry detergents (kg/cap/year).

Region	1970	2010	SSP1	SSP2	SSP3	SSP4	SSP5
North America	0.24	0.10	0.01	0.11	0.29	0.13	0.01
Central and South America	0.06	0.17	0.02	0.35	0.26	0.32	0.05
Middle East and Northern Africa	0.04	0.14	0.04	0.25	0.22	0.26	0.09
Sub-Saharan Africa	0.01	0.01	0.18	0.12	0.05	0.05	0.23
Western and Central Europe	0.22	0.05	0.01	0.08	0.27	0.08	0.01
Russia and Central Asia	0.07	0.12	0.03	0.15	0.24	0.23	0.06
South Asia	0.00	0.01	0.06	0.18	0.09	0.14	0.07
China Region	0.00	0.12	0.01	0.01	0.31	0.23	0.01
Southeast Asia	0.00	0.01	0.06	0.16	0.10	0.13	0.11
Japan and Oceania	0.11	0.18	0.01	0.01	0.36	0.15	0.01
Total	0.07	0.07	0.06	0.14	0.17	0.15	0.08

**Table 2:** Use of Phosphorus in dishwasher detergents (kg/cap/year).

Region	1970	2010	SSP1	SSP2	SSP3	SSP4	SSP5
North America	0.04	0.11	0.02	0.05	0.16	0.13	0.02
Central and South America	0.00	0.00	0.01	0.09	0.05	0.08	0.02
Middle East and Northern Africa	0.00	0.01	0.01	0.06	0.04	0.06	0.03
Sub-Saharan Africa	0.00	0.00	0.01	0.00	0.00	0.00	0.03
Western and Central Europe	0.03	0.10	0.02	0.06	0.16	0.13	0.02
Russia and Central Asia	0.00	0.01	0.02	0.04	0.06	0.07	0.03
South Asia	0.00	0.00	0.01	0.02	0.00	0.02	0.02
China Region	0.00	0.00	0.02	0.01	0.09	0.09	0.02
Southeast Asia	0.00	0.00	0.01	0.03	0.01	0.03	0.03
Japan and Oceania	0.02	0.11	0.02	0.02	0.18	0.12	0.02
Total	0.01	0.02	0.01	0.03	0.04	0.05	0.02

**Table 3:** Total emission of Phosphorus by laundry detergents (10^6^ kg P/year).

Region	1970	2010	SSP1	SSP2	SSP3	SSP4	SSP5
North America	69	45	3	69	159	73	4
Central and South America	13	83	11	211	180	186	24
Middle East and Northern Africa	6	51	23	154	149	162	49
Sub-Saharan Africa	3	12	283	209	111	98	349
Western and Central Europe	108	32	7	51	166	53	9
Russia and Central Asia	17	34	7	42	71	60	17
South Asia	2	22	125	417	258	319	142
China Region	4	167	6	7	410	277	7
Southeast Asia	1	5	41	122	82	98	75
Japan and Oceania	19	40	2	2	73	31	2
Total	241	491	509	1284	1658	1357	678

**Table 4:** Total emission of Phosphorus by dishwasher detergents (10^6^ kg P/year).

Region	1970	2010	SSP1	SSP2	SSP3	SSP4	SSP5
North America	11	51	12	28	88	70	14
Central and South America	0	2	8	54	34	47	13
Middle East and Northern Africa	0	5	7	35	28	38	16
Sub-Saharan Africa	0	0	17	9	4	6	45
Western and Central Europe	14	63	12	39	94	80	15
Russia and Central Asia	0	2	4	11	18	19	7
South Asia	0	0	20	50	1	36	34
China Region	0	2	22	17	121	106	25
Southeast Asia	0	1	10	25	11	22	22
Japan and Oceania	3	26	5	4	35	25	6
Total	29	153	116	271	434	450	198

These datasets were based on the use of detergents and the Phosphorus content in the detergents. The use of the detergents was based on the relation between income and detergent use. The content of Phosphorus in laundry and dishwasher detergents was based on the current levels and assumptions on the effect of environmental policy targets to reduce the Phosphorus load.

In this study, the five Shared Socio-economic Pathways (SSP) scenarios [2] are analyzed for their effect to the emissions of phosphorus from detergents. These scenarios differ in their population growth, economic growth and storylines.

## 2 Experimental design, materials and methods

### 2.1 Use of laundry and dishwasher detergents

**Fig. 1:**
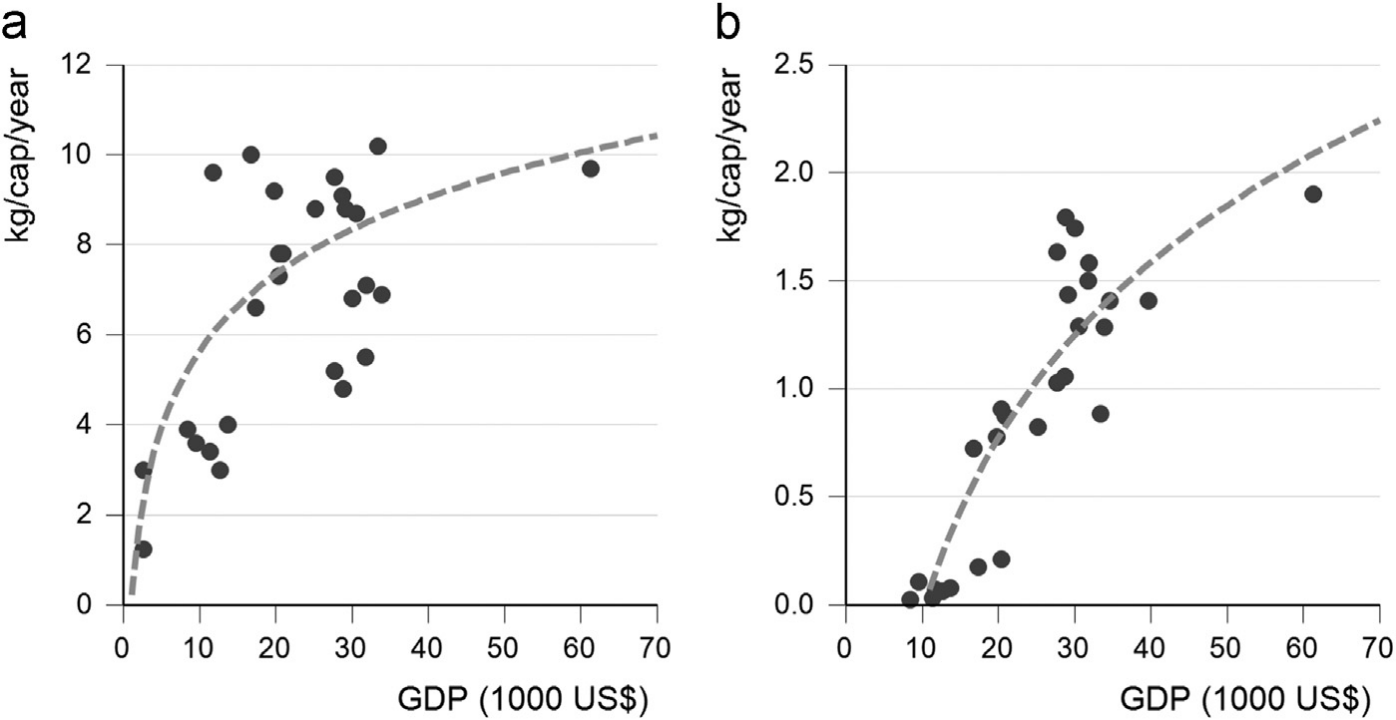
Use of laundry detergents (a) in EU Member States, Indonesia and China in relation to GDP and the use of dishwasher detergent in EU Member States (b) [4]. Formulas for the use of laundry detergents = 2.458 * ln (GDP) −17.445 (in kg cap^−1^ yr^−1^. *R*^2^ = 0.47), and for dishwasher detergents = 1.1738 * ln (GDP) – 10.852 (in kg cap^−1^ yr^−1^. *R*^2^ = 0.79).

The use of laundry and dishwasher detergents were related to the national GDP per capita (Fig. 1) [3]. We assumed a maximum use of laundry detergents of 10 kg cap^−1^ year^−1^ and a maximum use of 3 kg cap^−1^ year^−1^ for dishwasher detergents based on the historical maximum [4].

### 2.2 P content in laundry detergents

A minor but substantial share of P emissions are related to the use of laundry detergents in countries where a majority of households use washing machines. Before 1940, laundry detergents did not contain Phosphorus, but after 1945 the percentage of P in laundry detergents increased to between 6% and 8% or even more. The high loads of Phosphorus due to detergents resulted in eutrophic lakes. As a consequence, several countries set a maximum P content for detergents. In 1972, several US States set the maximum weight at 8.7%, but a few years later the first states reduced this to a maximum of 0.5% [5]. In the year 2000, 7 EU countries banned P-containing detergents [4]. In 2011, the EU declared a maximum content of 0.5 g P for a standard washing dose starting in 2013 [6].

The formula for the average Phosphorus content in laundry was:

(1) $$C_{L\det\_a}^{P}=C_{\mathrm{Lfree}}^{P}+\left(C_{L\det}^{P}-\;C_{\mathrm{Lfree}}^{P}\right)*f_{\mathrm{Lnormal}}^{P}$$

where by $C_{L\det\mathrm{\_a}}^{P}$ is the concentration of P in laundry detergents based on the mix of standard and P-free^1^ laundry detergents for a country; $C_{\mathrm{Lfree}}^{P}$ is the concentration of P in P-free detergents; $C_{L\det}^{P}$ is the concentration of P in normal laundry detergent; $f_{\mathrm{Lnormal}}^{P}$ is the fraction of use of standard laundry detergents related to the use of the total detergents for a country. The total emission of Phosphorus by laundry detergents is the combination of the detergent use and the Phosphorus content:

(2) $$E_{\mathrm{laundry}}^{P}=\left(g*\ln\left(\mathrm{GDP}\right)+h\right)*C_{L\det\_a}^{P}$$

where by, $E_{\mathrm{laundry}}^{P}$ is the emission of P from laundry detergents in kg cap^−1^ yr^−1^ in a country; and *g* is 2.458 and *h* is −17.445 (Fig. 1a).

We assumed a P concentration in laundry detergents of 0.0625 g P/g detergent for standard detergents and 0.0006 g P/g detergent for P-free detergents [7]. These values for P-free detergent were in accordance with the range of P concentrations in detergents in the United Kingdom [8]. The fraction of standard detergents $f_{\mathrm{Lnormal}}^{P}$ for countries for 2010 was based on the current situation [4], [6], [7]. For 1970, no P-free detergents were available.

### 2.3 P content in dishwasher detergents

Dishwasher detergents also had a high P content as no detergents without P were available until recently, when EU policy forced manufacturers to develop P-free detergents. According to EU policy, by 2018 dishwasher detergents cannot exceed 0.3 g P per washing dose [6]. To enable prognosis, the use of dishwasher detergents was related to GDP based on data of the year 2000 (Fig. 1b) [4].

The concentration and use of P in dishwasher detergents was calculated as follows:

(3) $$C_{D\det\_a}^{P}=C_{\mathrm{Dfree}}^{P}+\left(C_{D\det}^{P}-\;C_{\mathrm{Dfree}}^{P}\right)*f_{\mathrm{Dnormal}}^{P}$$

where by $C_{D\det\mathrm{\_a}}^{P}$ is the concentration of P in dishwasher detergents based on standard and P-free detergents; $C_{\mathrm{Dfree}}^{P}$ is the concentration of P in P-free detergents; $C_{D\det}^{P}$ is the concentration of P in standard dishwasher detergent; $f_{\mathrm{Dnormal}}^{P}$ is the percentage of use of standard dishwasher detergents in relation to use of total detergents. The total emission of Phosphorus by dishwasher detergents was:

(4) $$E_{\mathrm{dishwa}\mathrm{sher}}^{P}=\left(a*\ln(\;\mathrm{GDP}\;)+b\right)*C_{D\det\_a}^{P}$$

where by $E_{\mathrm{dishwasher}}^{P}$ is the emission of P from dishwasher detergents; and *a* is 1.1738 and *b* is −10.852 (Fig. 1b).

We assumed a P content of 0.117 g P/g detergent for standard detergents and 0.01 for P-free detergents [7]. These values for P content were in accordance with the range of P concentrations in detergents in the United Kingdom [8]. As P-free detergents were not widespread available in 2010, the fraction of standard detergents $f_{\mathrm{Lnormal}}^{P}$ for all countries was default 100% normal detergents [6].

### 2.4 Scenarios for the P content in detergents

Usage of P-free detergents was determined by the implementation of environmental policy measurements. In 2010, the use of P-free laundry detergents was mandatory in EU Member States, the United States, Japan and Singapore. P-free dishwasher detergents were hardly available in 2010. The Shared Socio-economic Pathways (SSPs) differ in environmental policy, from reactive environmental policy in SSP3 and SSP4, to proactive environmental policy in SSP1 and somewhere in between in SSP2. The use of detergents was modelled as a function on GDP and varied with the growth of the GDP between countries. The use of P-free detergents was based on GDP criteria:

• in SSP1, with strong environmental policy, all countries with a GDP of more than US$ 20.000 are projected to use P-free detergents by 2050;
• in SSP2, with moderate environmental policy, all countries with a GDP of more than US$ 40.000 are projected to use P-free detergents by 2050;
• in SSP3, with less environmental policy. traditional P-containing detergents are allowed; in countries where P-free detergents are currently in use, use of P-free is reduced by 50% in favorite of standard detergent by 2050;
• in SSP4, with less environmental policy, by 2050 that ratio will be between the current situation and that in SSP2;
• in SSP5, with moderate policy, all countries with a GDP of more than US$ 30.000 are projected to use P-free detergents by 2050.

We assumed standard detergents in all other situations and a gradual change of the fraction of P free detergents from the present-day up to 2050.

## Footnotes

1 P-free is used for detergents with a very low Phosphorus content, also called eco-detergents.
