# Supplementary material for: Datasets of the phosphorus content in laundry and dishwasher detergents
Source: Data Brief. 2018 Nov 19;21:2284–9. doi: 10.1016/j.dib.2018.11.081 (PMC6279942; doi:10.1016/j.dib.2018.11.081)
Supplement: Supplementary file 1 — Supplementary material [file mmc1.docx]

Den Hague, 26-11-2018

Dear madam, sir

Concerning the article *Datasets of the phosphorus content in laundry and dishwasher detergents*, there are no conflicts of interest between one of the authors and the results presented.

Yours sincerely,

Peter van Puijenbroek
